# Molecular Evolution of Immune Genes in the Malaria Mosquito *Anopheles gambiae*

**DOI:** 10.1371/journal.pone.0004549

**Published:** 2009-02-23

**Authors:** Tovi Lehmann, Jen C. C. Hume, Monica Licht, Christopher S. Burns, Kurt Wollenberg, Fred Simard, Jose' M. C. Ribeiro

**Affiliations:** 1 Entomology Branch, Division of Parasitic Diseases, Centers for Disease Control & Prevention, Chamblee, Georgia, United States of America; 2 Department of Biology, Emory University, Atlanta, Georgia, United States of America; 3 Laboratory of Malaria and Vector Research, National Institute of Allergy and Infectious Diseases (NIAID), National Institutes of Health, Rockville, Maryland, United States of America; 4 Bioinformatics and Computational Biosciences Branch, Office of Cyber Infrastructure and Computational Biology, National Institute of Allergy and Infectious Diseases (NIAID), National Institutes of Health, Bethesda, Maryland, United States of America; 5 Laboratoire de l'Instiut de Recherche pour le Developpement, RU#016, Organisation de lutte Contre les grandes Endemies en Afrique Centrale, Yaoundé, Cameroun; London School of Hygiene & Tropical Medicine, United Kingdom

## Abstract

**Background:**

As pathogens that circumvent the host immune response are favoured by selection, so are host alleles that reduce parasite load. Such evolutionary processes leave their signature on the genes involved. Deciphering modes of selection operating on immune genes might reveal the nature of host-pathogen interactions and factors that govern susceptibility in host populations. Such understanding would have important public health implications.

**Methodology/Findings:**

We analyzed polymorphisms in four mosquito immune genes (*SP14D1*, *GNBP*, *defensin*, and *gambicin*) to decipher selection effects, presumably mediated by pathogens. Using samples of *Anopheles arabiensis*, *An. quadriannulatus* and four *An. gambiae* populations, as well as published sequences from other Culicidae, we contrasted patterns of polymorphisms between different functional units of the same gene within and between populations. Our results revealed selection signatures operating on different time scales. At the most recent time scale, within-population diversity revealed purifying selection. Between populations and between species variation revealed reduced differentiation (*GNBP* and *gambicin*) at coding *vs.* noncoding- regions, consistent with balancing selection. McDonald-Kreitman tests between *An. quadriannulatus* and both sibling species revealed higher fixation rate of synonymous than nonsynonymous substitutions (*GNBP*) in accordance with frequency dependent balancing selection. At the longest time scale (>100 my), PAML analysis using distant Culicid taxa revealed positive selection at one codon in *gambicin*. Patterns of genetic variation were independent of exposure to human pathogens.

**Significance and Conclusions:**

Purifying selection is the most common form of selection operating on immune genes as it was detected on a contemporary time scale on all genes. Selection for “hypervariability” was not detected, but negative balancing selection, detected at a recent evolutionary time scale between sibling species may be rather common. Detection of positive selection at the deepest evolutionary time scale suggests that it occurs infrequently, possibly in association with speciation events. Our results provided no evidence to support the hypothesis that selection was mediated by pathogens that are transmitted to humans.

## Introduction

Infection in a susceptible host leads to parasite development or amplification, enabling disease transmission. In a resistant host, parasite development is halted. As pathogens that circumvent the host immune response are favoured by natural selection, so are host alleles that reduce parasite load. Such evolutionary processes leave their signature on the molecular makeup of the genes involved. In vertebrates, analysis of genetic diversity of the MHC (HLA in humans) genes showed that selection maintains exceptionally high allelic diversity [1]–[3]. Similar patterns were found in several members of the pathogen recognition encoding R gene family of plants [4]. Diversifying selection on these genes fits well with their known role in immune recognition, confirming that selection maintains “excess” (and ancient) alleles that differ in their capacity to recognize pathogens [5] by frequency dependent or overdominant balancing selection. If alleles conferring resistance to infection reduce the fitness of uninfected individuals, it is possible that balancing selection will maintain resistant and susceptible alleles as if they both conferred resistance to specific pathogens [4], [6]. An alternative scenario for host-pathogen interactions is the arms race [7], in which a series of selective sweeps alternate in pathogen and host populations, reflecting host genotypes that confer resistance and pathogen genotypes that facilitate infection. Selective sweeps reduce diversity within populations but enhance inter-population diversity. Unlike purifying selection, an arms race will be associated with a higher rate of substitutions that results in amino acid (aa) changes (K_A_) over that resulting in synonymous substitutions (K_S_) in alleles from different populations [8], [9]. Evidence for this form of positive selection has been found in surface antigens of many pathogens including *Plasmodium* spp. [1], [10], [11].

Molecular evolution of insect immunity genes has been studied primarily in *Drosophila*. Most studies have revealed weak evidence for adaptive evolution in general and especially in antimicrobial peptides [12]–[14]. Evidence of diversifying selection, as exemplified by the vertebrate MHC locus, was not found in these studies, and the arms race scenario was rarely supported. Studies on mosquito immune genes are in their infancy [15]–[18], and findings to date echo those on *Drosophila*. Understanding the forces and factors that govern pathogen susceptibility in host populations remain enigmatic [19]–[23] especially in arthropods whose innate immunity is thought to be their prime defense [24], [25]; many of which transmit pathogens to humans and domestic animals. Increased understanding of arthropod-pathogen relationships would have important public health implications for vector-borne diseases.

Recent advances in understanding the immune system of insect disease vectors have resulted in the identification of many genes whose products play key roles in these responses [26]–[29]. We selected four genes encoding molecules with different roles in the immune response mounted against eukaryotic and prokaryotic pathogens (Table 1). They include genes coding for *defensin*, *gram-negative bacteria-binding protein* (*GNBP*), a serine protease gene (*SP14D1*) and *gambicin*. These genes were implicated in *An. gambiae* responses to infection including with *Plasmodium* parasites (Table 1), although they probably do not include the main determinant locus of the mosquito natural susceptibility to malaria; which remains unknown to date.

**Table 1:** Location, basic structure, and function of selected genes.

Gene/Cytol^a^	Length/protein^b^	Immune Role (Pathogens)	Malaria response relevance
*SP14D1*	1,723 bp	Regulatory: signal transduction (Gram +ve, −ve bacteria, *Plasmodium*)	Distinguishes *A. gambiae* susceptible and resistant colonies [63]; localized at a resistance QTL -Pen3 [64]; upregulated after malaria infection [65]
2R∶14D1	360 aa (S_18_/P_91_/M_251_)		
*GNBP*	2,208 bp	Recognition (Gram −ve bacteria, *Plasmodium*)	Upregulated after malaria infection [26]
2R∶17C	396 aa (S_24_/M_372_)		
*Gambicin*	712 bp	Effector: antimicrobial protein (Gram +ve, −ve bacteria, Fungi, *Plasmodium*)	Upregulated after malaria infection; unique to culicidae; marginaly lethal to *Plasmodium berghei* [66]
3R∶30E	81 aa (S_18_/P_2_/M_61_)		
*Defensin*	1,410 bp	Effector: antimicrobial protein (Gram +ve, −ve bacteria, Fungi, *Plasmodium*)	Upregulated after malaria infection; [67], [68]; anti-Plasmodium activity [69]
3L∶41	102 aa (S_25_/P_37_/M_40_)		

a Cytological location of the gene. *AgSP14D1* is mapped in inversion 2Rd. The other three genes are outside polymorphic inversions.

b Total sequence length (bp) without deletions; total protein length (aa); length of signal peptide (S), cleaved propetide segment (P) and mature protein (M) in aa.

Here, we describe and decipher patterns of molecular variation at each gene within and between populations and sibling species of *Anopheles gambiae*, the principal vectors of malaria in Africa. We evaluate if different modes of selection shaped variation on these genes, and assess whether selection could be mediated by mosquito-transmitted human parasites i.e., selection by the protozoan agent of malaria, *Plasmodium falciparum* and the nematode agent of lymphatic filariasis, *Wuchereria bancrofti*. Here, we extend our limited study on *defensin* (Simard et al. 2007), while including the *defensin* data to enhance the scope of the current analysis. Comparing signatures of selection based on intra-population data, between conspecific populations and between sibling species, as well as between distant Culicid taxa (over 100 mya) might provide insights into the modes of selection operating on different time scales.

To evaluate selection mediated by “human” pathogens, we contrast patterns of molecular variation between anthropophilic vector (*An. gambiae s.s.* and *An. arabiensis*) and zoophilic non-vector (*A. quadriannulatus*) sibling species (Table 2). Similarly, we included four *A. gambiae* populations that differ in their exposure to human pathogens and span the range of geographical and genetic distances within this species e.g., [30]–[34]. For example, the transmission of *W. bancrofti* by *An. gambiae* and *An. arabiensis* is very high in Nigeria and moderate in eastern Kenya, but it is non-existent in western Kenya and Senegal (Table 2). Between-population variation in exposure to these pathogens is expected to correlate with selection pressure mediated by them. If selection mediated by human parasites dominated the evolution of a gene, we predict that divergence between anthropophilic species (*An. gambiae* and *An. arabiensis*) will be small in functional domains (e.g., exons), but high in neutral domains (e.g., introns) of the same gene, whereas, divergence between anthropophilic and zoophilic (*An. quadriannulatus*) species will be high across all domains. Likewise, we predict that patterns of within-gene differentiation between *An. gambiae* populations will be correlated with their exposure rate to human pathogens.

**Table 2:** Population characteristics in relation to exposure to human pathogens.

Species and Population	*An. qudriannulatus* Zimbabwe	*An. arabiensis* W. Kenya	*An. gambiae* W. Kenya	*An. gambiae* E. Kenya	*An. gambiae* Nigeria	*An. gambiae* Senegal
Date Collected	Jun. 1986	Jul. 1994	Jul. 1994	Aug. 1996	Jul. 1999	Aug. 1995
Method^a^	IR	IR-bednet	IR-bednet	IR	IR	HL
Sample size	14	13	12	11	14	10
Anthropophily^b^	Very low [71]	Moderate [71], [72]	High [31], [71], [72]	High [32], [71]	High [33], [71]	High [70], [71]
Local malaria transmission^c^	None [71]	Moderate 400 [31]	High 400 [31]	Low 10 [32]	Moderate 120 [73]	Moderate 260 [70]
Local filaria Transmission^d^	None [71]	None	None	Moderate [34]	High [33]	None

a Collection method included IR: Indoor-resting adult mosquitoes collected by pyrethrum-spray or aspiration; IR-bednet: blood fed and blood-seeking females collected by aspiration from net traps hung over the beds of sleeping volunteers; and HL: blood-seeking mosquitoes were collected by human landing catches.

b Refers to the mosquito preference to feed exclusively on human blood.

c Overall index of the intensity of malaria transmission measured as annual infective bites per person. Estimates reflect total transmission by all vector species because most studies identify *An. arabiensis* and *An. gambiae* as *An. gambiae* sensu lato.

d Overall index of the intensity of lymphatic filariasis transmission based on the prevalence of mosquito infected with larvae of *Wucheraria bancrofti*. None refers to locals where no clinical manifestations in people are known and no infected mosquitoes were found based on personal communication Frederic Simard (Senegal) and William Hawley (W. Kenya).

## Results

**Figure 1:**
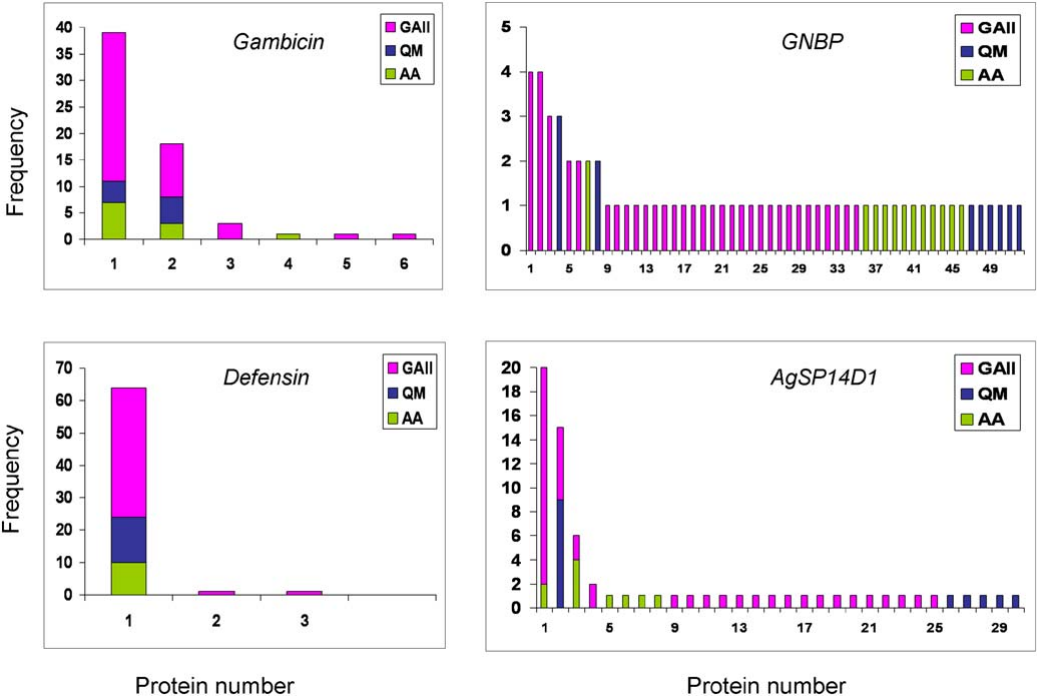
Mature protein (excluding signal peptide and the cleaved propetide segment) distribution within and between species.

Population characteristics are summarized in Table 2. Examination of protein variation might help delimit the modes of selection although it is less amenable for statistical tests. Therefore, variation in the mature protein (excluding signal peptide and cleaved domain, Table 1) is briefly described. No length variation was found across species in all proteins encoded by each gene. A single mature protein was shared across all three species in *defensin* (Figure 1, Table S1). The two common proteins found in gambicin (Figure 1, Table S1) were also shared across all three species. Two of the three common proteins of *SP14D1*, were shared between *An. gambiae* and *An. arabiensis* and one was shared between *An. gambiae* and *An. quadriannulatus* (Figure 1, Table S1). In *GNBP*, however, protein diversity was large (Figure 1, Table S1). Within populations, typically only one or two proteins had a frequency greater than one. Such common proteins were separated by only 1–2 aa changes from each other, whereas 1–3 aa changes separated all proteins from the most common one in that population (Table S1). With the possible exception of GNBP, these patterns are inconsistent with selection for hypervariability. Neutral evolution may explain protein variation in *gambicin*, *SP14D1*, and even in *GNBP*, because increased protein diversity in GNBP is expected, under neutrality, due to its length (Table S1). The lack of protein diversity across species in *defensin*, however, suggests that purifying selection is involved (Simard et al. 2007). Protein diversity in the zoophilic *An. quadriannulatus* showed no distinct features compared to those of the anthropophilic *An. gambiae* and *An. arabiensis*.

### Within Population Genetic Diversity

**Figure 2:**
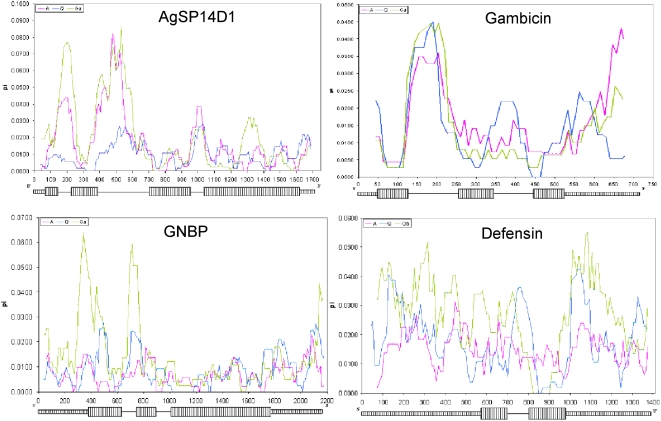
Polymorphism along the gene using sliding window (window length = 50 bp; sliding interval = 10 bp). Exons and flanking regions are denoted by broad and narrow hatched rectangles, respectively; introns are denoted by lines. A, Q, and Ga denote *An. arabiensis*, *An. quadriannulatus*, and *An. gambiae* from western Kenya, respectively.

**Figure 3:**
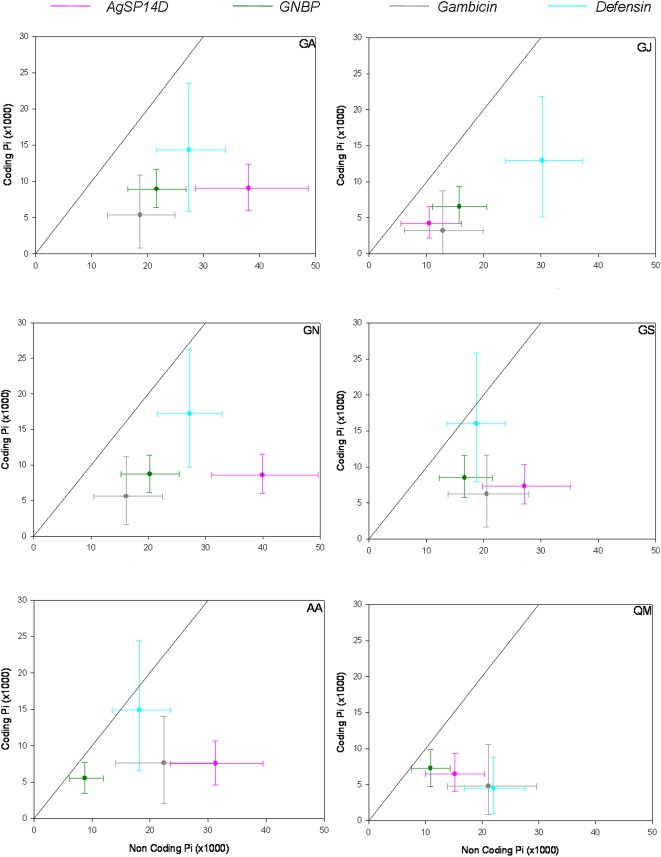
Diversity (π) and 95% CI in coding and NC regions in each population. Diagonal lines mark equal diversity of coding and NC regions. GA, GJ, GN, and GS denote *An. gambiae* populations from western and eastern Kenya, Nigeria and Senegal, respectively. AA and and QM denote *An. arabiensis* and *An. quadriannulatus* respectively.

A sliding window examination of nucleotide diversity across the genes revealed over a ten fold difference between maxima and minima of every species (Figure 2). Diversity in coding regions was significantly lower than that in non-coding regions for every gene in all populations (except *defensin* in *An. arabiensis* and *An. gambiae* from Senegal, Figure 3), in accordance with purifying selection. Diversity at non-coding (NC) regions differed significantly among genes (at all populations except *An. gambiae* from Senegal), but it did not predict among-gene diversity in coding regions, which did not differ significantly in any population (Figure 3). The correlation between recombination rates (between neighboring nucleotides) and nucleotide diversity in the coding region was not significant (r = 0.-19, P>0.38, df = 1/22), as was the total diversity (Table 3). High NC diversity and low coding diversity (e.g., *SP14D1*) is consistent with purifying selection, but where NC diversity is also low (e.g., *GNBP*, *gambicin*), positive selection, i.e., a recent selective sweep, cannot be ruled out.

**Table 3:** Nucleotide diversity (π×10^−3^), number of polymorphic sites (S), recombination parameter between adjacent position (R = 4Nr)×10^−3^, and ratio of nucleotide diversity in nonsynonymous/synonymous sites (ω = K_a_/K_s_) in coding regions in each population.

Pop^a^	*SP14D1*				*GNBP*				*gambicin*				*Defensin*				*Mean* c
	N	π/S	R	ω^b^	N	π/S	R	ω	n	π/S	R	ω	N	π/S	R	ω	ω
*W. Kenya*	12	20, 38	30	0.097	11	15, 46	79	0.22	10	12, 5	67	0.4	10	28, 10	21	0.25	0.24
*E. Kenya*	11	7, 18	42	0.16	11	11, 31	20	0.16	9	10, 2	81	u	11	27, 11	17	0.14	0.15
*Nigeria*	13	19, 40	8	0.18	10	14, 43	346	0.17	14	12, 6	994	0.31	12	26, 18	292	0.22	0.22
*Senegal*	10	15, 32	4	0.091	10	12, 40	84	0.17	10	15, 6	32	0.19	9	20, 13	37	0.12	0.14
*gambiae*	43	20, 91	10	0.12^***^	42	14, 119	148	0.17^***^	43	12, 13	87	0.40	42	27, 27	104	0.19^**^	0.22^a^ ^b^
*arabiensi*	11	16, 29	6	0.2^**^	13	7, 33	5	0.40	11	17, 7	62	0.40	13	15, 13	264	0.19^*^	0.30^a^
*quadrian*	14	9, 31	645	0.046^***^	11	9, 35	u	0.21^**^	10	15, 4	192	0.30	14	18, 6	1019	0.00^*^	0.14^b^
*Pooled* d	69	24, 135	20	0.12^b^	66	20, 168	131	0.26^a^ ^b^	64	15, 18	131	0.37^a^	69	31, 39	82	0.13^b^	0.22

a Populations of *An. gambiae* are referred by location and whereas, *gambiae*, *arabiensi*, and *quadrian*, represent *An. gambiae* (pooled), *An. arabiensis* and *An. quadriannulatus*, respectively.

b Testing equality of nucleotide diversity of synonymous and nonsynonymous sites (ω = 1) in coding regions was performed by using bootstrapping (see Materials & Methods) only at the species level. ^*, **, ***^represent P<0.05, P<0.01, and P<0.001 significance levels and u denotes undefined value.

c Average across genes for each population. Species values with different letter are statistically different from each other (P<0.05) as determined by Ryan-Einot-Gabriel-Welsch multiple range test following two way ANOVA of Nonsynonymous/synonymous diversity ratio over gene and species (separate *An. gambiae* populations were excluded).

d Pooled across populations (and sepecies) for each gene. Values with different letter are statistically different from each other as described above (c).

Under neutrality, a similar pattern of polymorphism is expected across functional regions. Comparing site frequency spectra between coding and non coding regions provided a comprehensive test of that variation. Frequency spectra were grouped into ‘rare alleles’ (singleton sites), ‘moderate alleles’ (sites where the rare nucleotide numbered two or three), and ‘common alleles’ (sites where the rare nucleotide was observed four or more times). Invariant sites were included to accommodate total length variation between regions. Contingency table analyses were used to assess the effect of functional region (coding vs NC), population, and their interactions on the frequency spectra. Within population differences in the polymorphism spectra between coding and NC regions were highly significant across all populations (P<0.01, Table 4). Heterogeneity χ^2^ tests showed no differences between populations (P>0.1) in all genes, providing no indication for local adaptation regardless of exposure to human pathogens, ie., comparing the zoophilic *An. quadriannulatus* with the anthropophilic *An. gambiae* and *An. arabiensis*. In coding regions, moderate and rare allele frequencies were particularly reduced (Table 4), as expected under purifying selection because it acts more strongly against rare polymorphisms, which include most deleterious mutations. Reduction in the frequencies of all allele classes (including common alleles) as detected in the coding regions of *SP14D1*and *GNBP* (Table 4) could indicate severe constraints or positive selection.

**Table 4:** Frequency spectra in coding (C) and non-coding (NC) regions across species at each gene.

Population	Region	*Def [C:306/NC:978–1016 nt]*				*SP14D [C:1083/NC:588–607 nt]*				*Gambic [C:243/NC:415–432 nt]*				*GNBP [C:1188/NC:930–959 nt]*			
		f = 0^a^	f = 1^a^	f = 2–3	f = 4–7	f = 0	f = 1	f = 2–3	f = 4–7	f = 0	f = 1	f = 2–3	f = 4–7	f = 0	f = 1	f = 2–3	f = 4–7
A. gambiae	Coding	96.7	0**	1.3*	2	97	2	0.7	0.7***	97.9	1.7*	0.4	na	96.1	3	0.6*	0.3
West Kenya	NoCod	91.4	3.1	4.2	1.3	90	3.4	1.7	5.2***	91.9	6.7	1.4	na	92.2	4.7	2.5**	0.6
*A. arabiens*	Coding	95.8	2	1.3	1	97	1.7*	0.7**	0.3	97.1	1.7	0.8	0.4	97.2	2.1	0.5	0.2
	NoCod	94.3	3.1	1.3	1.3	90	4.8**	3.8***	1.2	94	0.7	3.4	1.9	95.9	2.4	1.7*	0
*A. quad*	Coding	98	1.3*	0.3	0.3	97	1.9	0.4	0.6	98.4	1.2	0*	0.4	97.1	2.1	0.7	0.2
	NoCod	91.6	4.6	1.9	1.9	94	4.1*	1.3	1.1	93.4	2.3	3.5	0.5	95.4	3.4	0.7	0.4
*All (Pooled)*	Coding	96.1	1.4***	1.3**	1.3	97	2***	0.6***	0.4***	97.9	1.3**	0.5***	0.3	96.8	2.5	0.5***	0.2*
	NoCod	92	3.9*	2.6	1.5	91.4*	4.1***	2.4***	2.1***	93.5	3.6*	2.3*	0.7	94.5	3.1	1.8***	0.6*
	**Overall**	**93**	**3.3**	**2.3**	**1.5**	**96**	**2.7**	**1.2**	**0.5**	**95.1**	**2.8**	**1.6**	**0.5**	**95.6**	**2.7**	**1.1**	**0.4**

a Frequency spectra classes including invariant positions (f = 0), low polymorphism represented by singletons (f = 1), moderately polymorphic positions with the rare nucleotide observed twice or three times (f = 2–3), and highly polymorphic positions with the rare nucleotide observed four or more times (f = 4–7). The relative distribution of each class is expressed as percentages. Excess and deficit of observed vs. expected frequency is marked by red and blue respectively in cells with significant deviations based on 1 df χ^2^ test (^*^, ^**^, ^***^, represent P<0.05, 0.01, and 0.001, respectively). The western Kenya population of *A. gambiae* represents this species (heterogeneity χ^2^ test showed no evidence for heterogeneity among the four populations). All contingency tables for each gene and species were significant (P<0.01).

### Within Population variation in Synonymous and Nonsynonymous Sites

Diversity of nonsynonymous (K_A_) sites was lower than that of synonymous (K_S_) sites across species in all genes, although, it was not significantly lower in *gambicin* (and *GNBP* in *An. arabiensis*, Table 3). Heterogeneity among species in K_A_/K_S_ ratios was detected (Table 3; P<0.029, ANOVA, F = 6.8, df = 2/6), but contrary to expectations based on the degree of anthropophily, this ratio was higher in *An. arabiensis* than in *An. quadriannulatus* (*An. gambiae* was intermediate despite being most anthropophilic species). Heterogeneity among genes in K_A_/K_S_ ratios (Table 3; P<0.007, ANOVA, F = 11.0, df = 3/6) showed higher ratios in *gambicin* (across species). Higher K_A_/K_S_ ratio in *gambicin* may reflect elevated K_A_ due to the low intensity of purifying selection (relaxed constraints). However, K_A_ did not differ among genes (P>0.5, ANOVA, F = 0.6, df = 3/6) and *gambicin*'s K_A_ was ranked the second highest. To evaluate if K_S_ of *gambicin* was reduced, we used a covariance analysis regressing diversity in synonymous sites over diversity in nonsynonymous sites, species, and gene. Contrary to relaxed constraints, K_S_ of *gambicin* - was lower than that in all other genes (P<0.048, multiple least squares means comparison test). Since relaxed functional constraint does not account for these results, a better explanation is provided by negative balancing selection (see below).

### McDonald Kreitman Test

The McDonald Kreitman test (1991) compares the ratios of fixed to polymorphic substitutions of nonsynonymous (NS) and silent (both synonymous and NC) substitutions between species. These fixation rates are expected to be equal under neutrality, whereas positive selection is expected to increase the fixation rate in NS sites. The test could not be performed between *An. gambiae* and *An. arabiensis* because there were no fixed differences between them across all four genes (Table 5) in accordance with other evidence suggesting gene exchange (introgression) between them [46]–[48]. Departures from neutrality were detected only in *GNBP* in comparisons of both species with *An. quadriannulatus* (Table 5). In both cases, the ratios of fixed to polymorphic sites were lower in NS sites than those in silent sites. These results are inconsistent with positive selection operating by fixing different aa in each species at *GNBP*. Notably, the fixation rates of NS substitutions were not lower than those of other genes, as might be expected under purifying selection. Instead, the rates of fixation of silent substitutions were substantially higher than those of the other genes - as if positive selection operated on silent, rather than on NS substitutions in *GNBP*.

**Table 5:** McDonald Kreitman test (see text for details).

Gene G+C^a^	Pop^b^	Silent: (Fixed/Polymophic)	Nonsynonymous (Fixed/Polymorphic)	P
*SP14D1*	A-Q	0.075 (10/133)	0.095 (2/21)	Ns^c^
*0.59/0.54*	Ga-Q	0.068 (10/146)	0.100 (2/20)	Ns
*GNBP*	A-Q	0.351 (39/111)	0.028 (1/36)	**<0.001**
*0.58/0.51*	Ga-Q	0.191 (31/162)	0.00 (0/36)	**<0.01**
*Gambicin*	A-Q	0.056 (3/54)	0.00 (0/3)	Ns
*0.54/0.50*	Ga-Q	0.016 (1/61)	0.00 (0/2)	Ns
*Defensin*	A-Q	0.082 (12/147)	0.250 (1/3)	Ns
*0.62/0/51*	Ga-Q	0.018 (3/168)	0.00 (0/4)	Ns

a G+C content (over species) in the coding region/whole gene.

b The test could not be performed between *An. gambiae* and *An. arabiensis* because there were no fixed differences between them across all genes (see text for details).

c Not significant (P>0.05).

### Divergence/Differentiation Between Species and Populations

**Figure 4:**
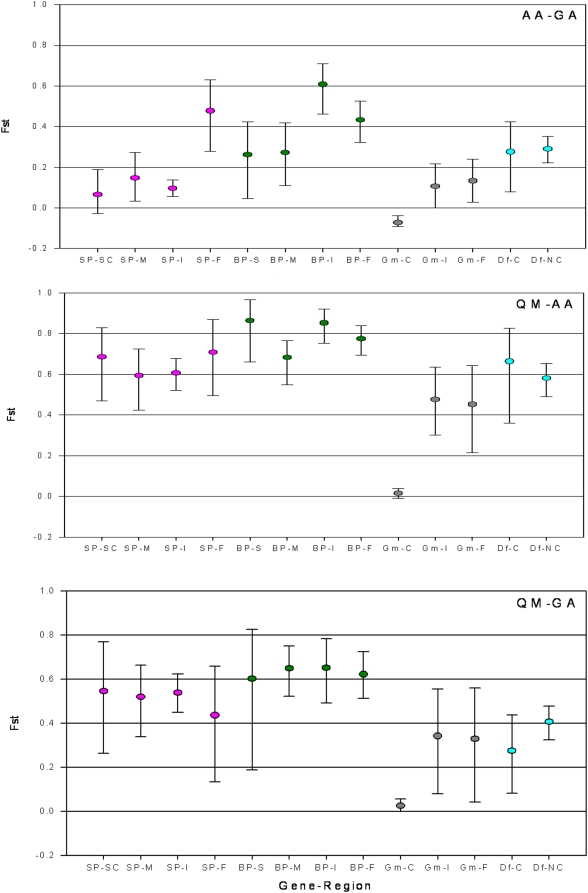
Divergence between species measured by F_ST_ in functional regions of each gene. The 95% CI were estimated by bootstrapping over positions (1000 bootstrap replications) provided that there were ten or more variable positions in that region across the pair of populations compared. *An. gambiae* is represented by its western Kenya population (GA). *Defensin*, *gambicin*, *GNBP*, and *SP14D1* are denoted by Df, Gm BP, and SP, respectively. NC denotes noncoding regions, C denotes coding regions, F denotes flanking regions, I denotes intronic region, M denotes mature protein, and SC, denotes signal and cleaved propetdide segment.

Within-gene heterogeneity in divergence, measured by F_ST_, is evidence for selection [52]. Heterogenic differentiation across functional domains of the same gene were observed in five out of twelve tests (P<0.05 in individual test, and Binomial multiple test: P<0.0002). In all comparisons, divergence in coding regions (including all polymorphic sites) was lower than that in NC regions. The most pronounced heterogeneity was observed in *gambicin* across all three species pairs, but a similar, less extreme pattern was found in *GNBP*, and in *SP14D1*, between *An. arabiensis* and *An. gambiae* (Figure 4). Zero divergence (F_ST_ = 0) in the coding region of *gambicin* as opposed to its high divergence in intronic and flanking regions (F_ST_>0.4, Fig 4) is remarkable, given that the polymorphism in the coding region was comparable to other genes (Table 3 and 4). The divergent “haplotype” of synonymous mutations and the distinct introns across species (not shown) do not support positive selection driving one allele across all three species.

Despite the dramatic within-gene heterogeneity in divergence observed in *gambicin*, HKA tests [42] between coding and noncoding regions were not significant in all four genes. Insignificant results persisted even when the average number of substitutions per site between species (Dxy) in the coding region was set to zero, indicating that the test had low power [8].

**Figure 5:**
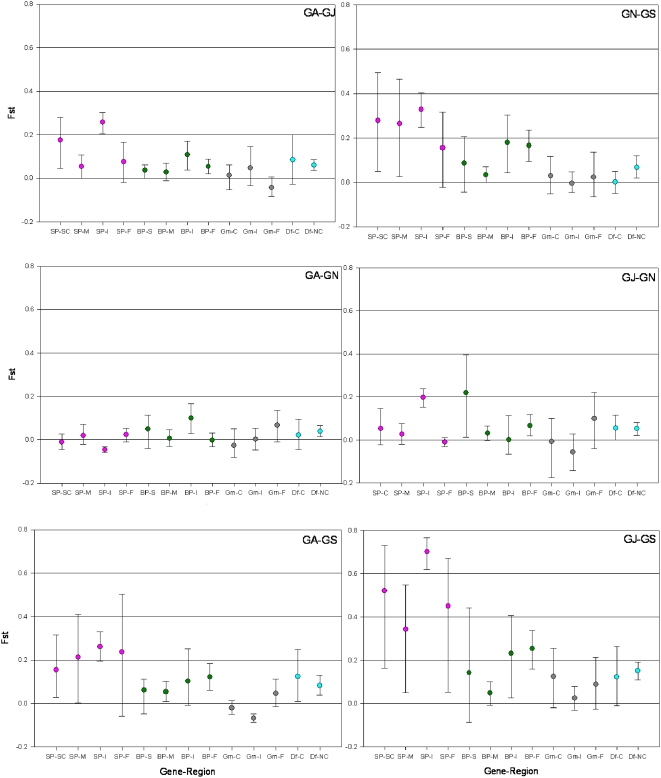
Differentiation between An. gambiae populations measured by F_ST_ in different functional regions of each gene. The 95% CI of each value were estimated by bootstrapping over positions (1000 bootstrap replications) provided that there were five or more variable positions in that gene segment across the pair of populations compared. The number of variable positions is shown if it is below 10. Horizontal axis legend is the same as in Figure 4.

Within-gene heterogeneity in differentiation between *An. gambiae* populations, was detected in five out of 24 tests (Figure 5; P<0.05 in individual test, and Binomial multiple test: P<0.006). Heterogeneity across functional domains of the same gene were observed in *SP14D1* (3 tests) and *GNBP* (2 tests), whereas differentiation in *gambicin* was minimal across its functional regions. In genes showing heterogeneity, differentiation in coding region(s) was lower than corresponding NC region(s), as expected under balancing selection. Contrary to predictions (see Introduction), differentiation heterogeneity pattern was not correlated with population exposure to human pathogens. Instead, it appears to be correlated with the overall magnitude of differentiation between populations, probably reflecting higher power to detect heterogeneity when expected (neutral) differentiation is high.

### Selection During Culicidae Evolution

Tests of positive selection were performed using the codeml program in the package PAML 3.15 [49] based on gene trees of members of the Culicidae. Counting nonsynonymous and synonymous substitutions separately in every codon along the branches of the tree, the likelihood of positive selection (ω>1, where ω = K_A_/K_S_) is estimated allowing for heterogeneity in the mode and intensity of selection among codons. Considering that the time of divergence between the Culicinae and the Anophelinae exceeds 100 my [53], this analysis was aimed at evolutionary changes that occurred on a considerably “deeper” time scale than previous analyses, based on variation within and between populations of sibling species.

Positive selection was not detected for *Defensin*, *GNBP* and *SP14D1* (Table 6). Strong evidence for positive selection, however, was found at *gambicin*, where ω exceeded 11 at one codon (codon 72, Table 6). Six variants of the mature protein were observed among 64 sequences representing members of the *An. gambiae* complex and three of these variants were common (frequency>2, Figure 1). All three common proteins had substitutions in the same codon. Phenylalanine and valine were shared by all three members of *An. gambiae*, whereas isoleucine was found only in *An. gambiae*. *An. funestus* had a similar nonpolar aa – leucine. Unlike these variants, formed by conservative substitutions, *An. darlingi* shared the polar aa tyrosine with *Culex pipiens* (and *Cx. quinquefasciatus*), whereas *Aedes aegypti* and *Armigeres subalbatus* had alanine in this site. Amino acid diversity in this site was exceptionally high both within *An. gambiae* and between distant taxa, but reversal mutations were not common.

**Table 6:** Positive selection on single codon level based on PAML (see text for details).

Gene^a^	Models^b^	ωS^c^	p(ωS)^d^	−2ΔLL^b^	P^b^	aa^e^
*GNBP*	M1 vs. M2	1	9.1	0	Ns	None
*GNBP*	M7 vs. M8	1	3.8	5.9	Ns	None
*SP14D1*	M1 vs. M2	1	1.1	0	Ns	None
*SP14D1*	M7 vs. M8	1.97	2.4	6.6	0.037	206^ns^; 169^ns^
*Gambicin*	M1 vs. M2	12.1	1.3	6.4	0.041	72^**^
*Gambicin*	M7 vs. M8	11.1	1.3	11.8	0.001	72^**^
*Defensin*	M1 vs. M2	1.4	0	1.1	Ns	None
*Defensin*	M7 vs. M8	2.2	2.4	0.9	Ns	None

a *GNBP* alignment was 171 aa long and included eight species; *SP14D1*alignment was 246 aa long and included six species; *Gambicin* alignment was 81 aa long and included nine species; *Defensin* alignment was 101 aa long and included seven species (see Materials and Methods for the species listing for each gene).

b Likelihood ratio tests (with 2 df) were used to determine the significance of finding ω>1 over all codons by comparing selection models (M2 and M8) that allowed for ω>1 with neutral (M1 and M7) models that allowed only ω≤1.

c Estimate of the highest ω value for any codon.

d The proportion of codons with the highest w estimate.

e Positions of the amino acids with ω>1 and their significant value estimated by BEB test in PAML.

## Discussion

Variation in the susceptibility to pathogens in insects and to malaria parasites in mosquitoes has been amply demonstrated [27], [54]–[57] and immunity factors have been repeatedly linked to the variation in susceptibility [26]–[29]. *Drosophila* innate immune genes diverged between species (on average) faster than non-immune genes but no evidence for positive balancing selection maintaining higher protein diversity (hypervariability) has been found by most studies [13], [14], [58], [59]. In addition, only a few examples of positive selection have been described [12], [14], [60], providing support for the arms race or the diversifying selection models of insect-pathogen interactions. Similarly, recent studies on mosquitoes detected only faint signals of positive selection or none [15]–[18].

We described and analyzed polymorphisms in four mosquito immune genes to decipher selection effects, presumably mediated by pathogen-mosquito interactions. Inference on selection relied on within-gene heterogeneity i.e., in synonymous vs. non-synonymous substitution rates. Within-gene heterogeneity is not confounded by factors such as demographic history, introgression, shared ancestral polymorphism and inversions which are known to confound comparisons between genes. Focusing the analyses on different taxonomic units afforded the opportunity to examine processes that have shaped genetic variation at several evolutionary time scales. Our main results can be summarized as follows. At the most contemporary time scale, probed by within-population variation, purifying selection alone was detected. At a deeper time scale, probed by between populations and sibling species variation, signatures of negative frequency-dependent balancing selection were detected on two (maybe three) genes. At the deepest time scale, spanning anopheline evolution, positive selection was detected on a single gene - *gambicin*. Our evidence does not support the hypothesis that selection was mediated by pathogens that are transmitted to man.

At the most contemporary time scale, intra-population polymorphisms revealed ample evidence for purifying selection on all genes. This evidence included lower diversity in coding vs. NC regions, a deficit of rare and moderate frequency SNPs at the coding regions, and K_A_/K_S_ ratios below one across all populations. An inconclusive signal of negative balancing selection was detected on *gambicin* by an elevated K_A_/K_S_ ratio (0.4, not statistically lower than one) due to reduced K_S_.

At a slightly longer time scale, intra-species variation revealed reduced differentiation at the mature protein compared with the same gene's NC regions (*GNBP* and *SP14D1*). Within-gene heterogeneity among functional regions in differentiation in both genes persisted at inter-species level between *An. gambiae* and *An. arabiensis*. Such heterogeneity cannot be explained by variations in mutation, recombination, introgression, or shared ancestral polymorphism because these effects are unlikely to be divided among functional domains of the same gene. Given considerable polymorphism within-populations (Tables 3 and 4), purifying selection poorly explains the observed pattern because it affects polymorphism and divergence rather than divergence alone. Correspondingly, the same significant pattern was obtained by bootstrapping the average number of substitutions per site (Dxy, not shown). The observed pattern is better explained by balancing selection on coding regions [52] regardless if the selection operated before or after speciation (see below about alternative explanations). Patterns of divergence between sibling species, extending the time scale of analysis, showed remarkable heterogeneity among functional regions of *gambicin* across all three species pairs, with over ten fold reduced divergence in coding as opposed to NC regions. Likewise, frequency dependent (negative) balancing selection provides a compelling explanation for the MK test on GNPB between *An. quadriannulatus* and both *An. gambiae* and *An. arabiensis*, showing high rate of fixation of synonymous substitutions. Accordingly, the aa under selection remain protected from loss because selection increases their frequency as they become rare, but consequent fluctuations in protein frequencies increase drift and fixation of partially linked silent substitutions. *GNBP*'s high protein diversity and its role in pathogen recognition fit well with this explanation. Nonetheless, positive selection on silent substitutions affecting transcription and expression cannot be ruled out, although it is unlikely.

Whether these results can be more parsimoniously explained by neutral or purifying selection needs to be addressed, especially because the HKA test, applied to coding and NC regions of each gene detected no significant results. Notably, the HKA test considers independent genealogy for each “gene”, even though this does not apply for exons and introns of the same gene. Thus, it appears to be overly conservative for within gene testing. Clearly, significant heterogeneity in differentiation and divergence among functional regions of the same gene cannot be reconciled with a neutral explanation. Purifying selection due to functional constraints limits variation in coding regions by removing deleterious mutations. Hence, it limits both polymorphism and divergence, but the fewer neutral (e.g., synonymous) or minimally deleterious mutations that attain moderate or high frequencies are subject to drift – similarly to mutations in NC regions. Therefore, unless polymorphism in the mature protein is near zero, purifying selection primarily limits the number of polymorphic sites, whilst drift continues to shape differentiation and divergence as it does for neutral loci. Strong purifying selection might even increase drift in coding regions and so, elevate differentiation due to smaller effective population size. Because polymorphism in the coding regions was not exhausted as our data showed, purifying selection cannot explain the ten fold reduced divergence in coding as opposed to NC regions at *gambicin*. In other words, why has the strong drift on NC regions (F_ST_>0.4) not fixed the common multiple proteins shared across species? Likewise it cannot explain why heterogeneity in divergence was not observed in *defensin* despite being subjected to purifying selection more than the other genes as indicated by finding a single mature protein across all species (see also Simard et al. 2007).

At the longest time scale, spanning over 100 my of Culicidae evolution [53], [61], PAML analysis detected strong positive selection on *gambicin*. At a single codon, nonsynonymous mutations occurred at a rate over 10 fold higher than the rate of synonymous mutations. No evidence for positive selection was detected in the other genes.

Consistent with previous studies on vectors, our results confirm that purifying selection is the most common mode of selection operating on immune genes [15]–[18] as it operated on all genes at the contemporary time scale. Signatures of negative frequency-dependent balancing selection were detected at least on *gambicin*, and *GNBP* during recent evolutionary time scales, suggesting that a diverse (maybe fluctuating) body of pathogens mediate balancing selection to maintain several alleles of immune genes. Positive selection was detected at the longest time scale spanning over 100 my on *gambicin*, suggesting that an arms race occurs rather rarely in accord with previous studies that detected no positive selection on recent evolutionary time scales [15]–[18]. Positive selection may be associated with speciation events following exposure to new pathogens. The low specificity of an innate system faced with myriad targets may constrain evolution of immune genes because enhanced defense against one pathogen may reduce defense against another [23], [62]. Clearly, such interpretations based on an exploratory investigation using four genes and a few species are merely tentative. These results add to the growing body of studies on immune genes of vector species that found little evidence for positive or classical diversifying selection [15]–[18] and of other insects [13], [14], [58], [59].

Finally, our results do not support the view that selection on these genes was mediated by human pathogens because overall, patterns of genetic variation are homogenous across the zoophilic *An. quadriannulatus* and the anthropophilic *An. gambiae* and *An. arabiensis* as well as across population of *An. gambiae* that differ in their exposure to human pathogens. Contrasting these results with corresponding patterns from the gene(s) that confer resistance to human pathogens might provide useful insights on *Plasmodium*-vector interactions. Identification of such gene(s) appears to be very near.

## Materials and Methods

### Mosquito Samples

*Anopheles gambiae* mosquito collections were made between 1994 and 1999 (Table 2). Collection sites include Asembo Bay in western Kenya, Jego in eastern Kenya, Gwamlar in central Nigeria, and Barkedji in Senegal. For brevity, population names used hereafter are western and eastern Kenya, Nigeria, and Senegal, respectively. *An. arabiensis* specimens were collected in Asembo Bay. *An. quadriannulatus* DNA was kindly provided by F. H. Collins and Nora Besansky from specimens collected in a rural area of southern Zimbabwe in 1986 [35]. At each site, mosquitoes were collected within one period from houses less than 5 km apart. Further details are found in Lehmann et al. [30].

### DNA extraction, species identification, and sequencing

Anopheline mosquitoes were visually identified as members of the *An. gambiae* complex. Genomic DNA was extracted from whole mosquitoes as described previously [30] and suspended in 100 µl of TE. Species identification was carried out using the PCR assay [36]. Molecular form of the *An. gambiae* specimens was determined using the PCR-RFLP assay [37]. *An. gambiae* specimens collected from Kenya and Nigeria were all of the S form, while those from Senegal were of the M form. PCR reactions to amplify the full target gene were carried out using 2 µl of template DNA (from an aliquot of whole-mosquito extracts diluted 1∶20 in distilled water) in 50 µl reaction containing 5 units Taq polymerase (Boehringer Mannheim or Gibco BRL) in manufacturer's buffer, 1.5 mM MgCl_2_, 200 µM each dNTP (PE Applied Biosystems) and 50 pmol each forward and reverse primers. To minimize PCR errors, amplification of *SP14D1* and *GNBP* were performed using a mixture of Taq polymerase and (Pfu Promerga) mixed 1∶7, respectively. Amplification of *Gambicin* was performed using Pfu only.

Primers were designed based on the published sequence of each gene. Cycling conditions for amplification included denaturation at 94°C for 5 minutes, followed by 35 cycles at 94°C for 30 seconds, 52°C for 30 seconds and 72°C for 1 minute, with a final extension step at 72°C for 5 minutes. PCR products were examined on a 1% agarose gel, and cloned using the pGem T-vector kit (Promega). Individual transformed colonies (white) were selected. The size of the DNA insert was determined by PCR using pUC/M13 forward and reverse primers. In most cases, a single appropriately sized insert was chosen at random, and sequenced in both directions after purification with the Wizard PCR Purification Kit (Promega). In addition to the previous forward and reverse primers, internal nested primers were used as sequencing primers. Cycle sequencing was performed using PE BigDye Terminator Ready Reaction Kit according to manufacturer's recommendations (PE Applied Biosystems). Sequencing reaction products were analyzed on an ABI 377 sequencer (PE Applied Biosystems). Sequences were checked for accuracy on both strands using Sequence Navigator (PE Applied Biosystems). Multiple alignments were performed with the Pileup program of GCG (Genetics Computer Group, 1999) using default options, and were adjusted by eye. To avoid sampling bias, a single allele (haplotype sequence) was arbitrarily selected from each specimen for the analysis. Alignments of variable positions are provided in supporting information figures (Figure S1, S2, S3, S4). DNA sequences have been deposited in GenBank (*Defensin* sequences have been deposited under the accession numbers DQ211988–DQ212056; *Gambicin*, *GNBP*, and *SP14D1* were deposited under accession numbers FJ653713–FJ653911).

### PCR error

Because multiple insertion/deletion (indels) were common in *SP14D*, *GNBP* and *defensin*, direct sequencing was not possible. Sequences were determined from 2–4 independent clones of the same allele, to identify errors resulting from mis-incorporation of nucleotides by Taq polymerase during the PCR amplification. We estimated PCR error rate to be 0.001 per bp in accordance with published records (Kwiatowski *et al.*, 1991). High variation between alleles, allowed distinguishing different alleles and different clones of the same allele. *Gambicin* was amplified using Pfu only, which practically eliminates PCR errors. Few indels in *gambicin* facilitated direct sequencing, which was used to verify sequences derived from clones (as above).

Although we used statistics that are less sensitive to the effect of PCR errors (e.g., nucleotide diversity instead of the number of segregating sites and theta derived based on the latter), the polymorphism reported here is slightly biased upwards because of PCR errors. Nevertheless, our inference is unbiased because instead of relying on the absolute values of polymorphism, we compared polymorphism between different functional regions of the gene that have the same probability to include a PCR error once differences in sequence length were accommodated (below).

### Data analysis

Nucleotide diversity (π) was estimated using DnaSp 4.10 [38]. The 95% confidence interval (CI) of π was estimated using bootstrapping over positions in programs written in SAS (SAS Institute Inc., 1990). To evaluate if recombination rate differed between genes and determined their diversity the recombination parameter (R = 4Nr) between adjacent nucleotide positions for each gene was estimated using DnaSp. A more complete summary of polymorphism was obtained by the site frequency spectra [39], [40], which describes the frequency of sites that are invariant (f = 0), singleton (f = 1), and polymorphic (f = 2, 3, … n/2), where f is the frequency of the rare nucleotide at this site/position and n is the number of sequences. These spectra distinguish between rare (e.g., singletons) and common mutations (sites where the rarest nucleotide was observed 4–7 times, which is the maximum possible frequency given 9–14 sequences per population). The frequency of neutral mutations increases slowly compared with positively selected mutations but faster than deleterious mutations. Hence, rare mutations represent a greater fraction of new and mildly deleterious mutations, whereas common ones represent a greater fraction of ancient and neutral mutations. The site frequency spectrum is especially useful to compare polymorphism in different regions of a gene without bias due to PCR errors, because it accounts for sequence length variation. We compared and tested equality of nucleotide diversity of synonymous and nonsynonymous sites using bootstrapping in MEGA 3.1 [41].

The Hudson, Kreitman and Aguadé's test (HKA test) compares within and between species divergence and polymorphism in two (or more) loci, accommodating different rate of neutral polymorphism between loci [42]. This test was designed to detect positive and positive-balancing selection. It was performed using DnaSP. The McDonald and Kreitman's Test (1991) compares the ratios of fixed to polymorphic substitutions of nonsynonymous and silent (both synonymous and NC) substitutions between species. Under neutrality, fixation rate is expected to be equal, but positive selection would increase the rate of fixation in nonsynonymous sites. This test was performed using DnaSP.

Differentiation between populations was assessed by sequence-based F statistics analogous to Wright F statistics [43], calculated according to [44] and tested (for being greater than zero) by a permutation test using DnaSP. Confidence intervals around F_ST_ values were calculated by bootstrapping over nucleotide positions using programs written in SAS [45]. To avoid the effect of unequal sample size due to pooling four *An. gambiae* populations compared with single populations of *An. arabiensis* and *An. quadriannulatus*, inter-species comparisons were performed using the population of *An. gambiae* from western Kenya, which is sympatric with *An. arabiensis*. The binomial test (which estimates the probability of obtaining the observed number of significant tests at the 0.05 level given the total number of tests) was used to detect significant departures from null hypothesis across multiple tests, such between pairwise population comparisons across genes.

The evolutionary relationship between the sibling species is not fully resolved probably because introgression between *An. gambiae* and *An. arabiensis* affected genes unprotected by fixed inversions [46]–[48]. Because of uncertain phylogeny and introgression, we did not classify mutations as ancestral, shared, and derived and our selection analysis relied on within-gene comparisons. Comparisons between different functional regions of a gene (defined below) and synonymous vs. non-synonymous mutations provide robust evidence for selection and avoid confounding effects of population demography, inversion, introgression, and PCR errors because they affect all regions of the gene equally. Likewise, such comparison is not susceptible to variation in mutation and recombination rates between unlinked loci across the genome. This approach is conservative because polymorphism in shorter DNA fragments is subject to higher sampling variation, reducing the power to detect differences between regions. Physical linkage between adjacent regions may further reduce the differences between them even if selection operated on only one region. The advantage of this approach, however, is that significant differences represent robust evidence for selection.

Test of positive selection on single codons was performed using the codeml program in the package PAML 3.15 [49]. It estimates the per site ratio of nonsynonymous to synonymous substitutions in every codon along the branches of a phylogenetic tree by fitting nested maximum likelihood models with different parameters. Analyses were performed on coding regions of all homologue genes from the family Culicidae available in Genbank (searched using tblastx) and all unique sequences obtained in this study. *GNBP* alignment was 171 aa long and included eight species (*An. gambiae*, *An. arabiensis*, *An. quadriannulatus*, *Ae. aegypti*, *Ae*, *albopictus*, *Ae. triseriatus*, *Cx.quinquefasciatus*, and *Armigeres subalpatus*). *SP14D1* alignment was 246 aa long and included six species (*An. gambiae*, *An. arabiensis*, *An. quadriannulatus*, *Ae. aegypti*, *Cx.quinquefasciatus*, and *Ar. subalpatus*). *Gambicin* alignment was 81 aa long and included nine species (*An. gambiae*, *An. arabiensis*, *An. quadriannulatus*, *An. funestus*, *An. darlingi*, *Ae. aegypti*, *Cx.quinquefasciatus*, *Cx.pipens*, and *Ar. subalpatus*). *Defensin* alignment was 101 aa long and included seven species (*An. gambiae*, *An. arabiensis*, *An. quadriannulatus*, *An. funestus*, *An. darlingi*, *Ae. aegypti* and *Ar. subalpatus*). Multiple alignment of coding regions was done using ClustalW [50] followed by hand alignments before removal of all gaps. For *GNBP* and *SP14D*, pairwise local alignment were obtained in tblastx instead of Clustal and final alignment was performed manually in Genedoc (version 2.700). Neighbor Joining trees were produced using the program Neighbor (PHYLIP 3.66) based on a distance matrix computed by Dnadist (PHYLIP 3.66), run under default parameters [51].

## Supporting Information

Table S1 Within population protein diversity (mature protein only)
(0.06 MB DOC)

Figure S1 Alignment of polymorphic positions in gambicin after exclusion of all gaps (indels). Dots indicate identity with corresponding base of the first sequence. Position number is indicated above each base and species affiliation on the left of each sequence. Silent changes in coding regions are highlighted in gray and amino acid replacement changes are highlighted in red. Note the two replacement mutations in nucleotide position 503 (see text for details).
(0.11 MB XLS)

Figure S2 Alignment of polymorphic positions in AgSP14D1 after exclusion of all gaps (indels). Dots indicate identity with corresponding base of the first sequence. Position number is indicated above each base and species affiliation on the left of each sequence. Silent changes in coding regions are highlighted in gray and amino acid replacement changes are highlighted in red
(0.30 MB XLS)

Figure S3 Alignment of polymorphic positions in Defensin after exclusion of all gaps (indels). Dots indicate identity with corresponding base of the first sequence. Position number is indicated above each base and species affiliation on the left of each sequence. Silent changes in coding regions are highlighted in gray and amino acid replacement changes are highlighted in red
(0.30 MB XLS)

Figure S4 Alignment of polymorphic positions in GNBP after exclusion of all gaps (indels). Dots indicate identity with corresponding base of the first sequence. Position number is indicated above each base and species affiliation on the left of each sequence. Silent changes in coding regions are highlighted in gray and amino acid replacement changes are highlighted in red
(0.38 MB XLS)
