# Supplementary material for: Molecular Evolution of Immune Genes in the Malaria Mosquito Anopheles gambiae
Source: PLoS One. 2009 Feb 23;4(2):e4549. doi: 10.1371/journal.pone.0004549 (PMC2642720; doi:10.1371/journal.pone.0004549)
Supplement: Table S1 — Within population protein diversity (mature protein only) (0.06 MB DOC) [file pone.0004549.s001.doc]

Table S1. Within population protein diversity (mature protein only)

| Gene, Length | Parameter | A. arabiensis | A. quadriannul | gamAB | gamJO | gamNG | gamSN | All pooled |
| --- | --- | --- | --- | --- | --- | --- | --- | --- |
| *GNBP* | h (A/S/n)^a^ | 99% (12/11/13) | 93% (8/6/11) | 98% (10/9/11) | 98% (10/9/11) | 98% (9/8/10) | 98% (9/8/10) | 99% (53/45/66) |
| 372 aa | Peptype -max freq^b^ | F - 15% | B - 27% | A - 18% | C - 18% | E - 20% | D - 20% | A - 6% |
|  | mean-dif (maxd)^c^ | 3.7 (7) | 2.6 (6) | 3.6 (6) | 2.8 (6) | 3.9 (7) | 3.4 (5) | 3.9 (9) |
| *SP14D* | h (A/S/n) | 84% (6/4/10) | 60% (6/5/14) | 85% (8/7/12) | 62% (5/4/11) | 91% (9/8/12) | 64% (4/2/10) | 87% (30/26/69) |
| 251 aa | Peptype -max freq | C - 40% | B - 64% | A- 42% | A - 64% | A - 33% | B - 60% | A - 29% |
|  | mean-dif (maxd) | 1.7 (6) | 0.7 (2) | 1.3 (3) | 0.9 (3) | 2.3 (5) | 1.2 (4) | 1.8 (7) |
| Gambicin | h (A/S/n) | 56% (3/1/11) | 56% (2/0/9) | 47% (2/0/10) | 81% (4/1/9) | 57% (4/2/14) | 0.2 (2/1/10) | 55 (6/2/64) |
| 61 aa | Peptype -max freq | A - 64% | B - 56% | A- 70% | A-B - 30% | A - 64% | A - 90% | A - 61% |
|  | mean-dif (maxd) | 0.7 (2) | 0.55 (1) | 0.5 (1) | 0.9 (2) | 0.6 (2) | 0.2 (1) | 0.6 (3) |
| Defensin | h (A/S/n) | 0% (1/0/10) | 0% (1/0/14) | 0% (1/0/10) | 18% (2/1/11) | 17% (2/1/12) | 0% (1/0/9) | 0% (3/2/69) |
| 40 aa | Peptype -max freq | A - 100% | A - 100% | A - 100% | A - 91% | A - 92% | A - 100% | A - 100% |
|  | mean-dif (maxd) | 0 (0) | 0 (0) | 0 (0) | 0.2 (1) | 0.2 (1) | 0 (0) | 0 (0) |

^a^ h: haplotype diversity; A: number of distinct alleles; S: number of singletons; n: number of sequences.

^b^ The relative frequency (%) of the most common protein; different letters indicate different proteins.

^c^ Mean number of aa differences between pairs of proteins; (maximum number of aa differences between pairs of proteins);
